# Reactivations after 5-methoxy-N,N-dimethyltryptamine use in naturalistic settings: An initial exploratory analysis of the phenomenon’s predictors and its emotional valence

**DOI:** 10.3389/fpsyt.2022.1049643

**Published:** 2022-11-29

**Authors:** Ana María Ortiz Bernal, Charles L. Raison, Rafael L. Lancelotta, Alan K. Davis

**Affiliations:** ^1^Department of Human Development and Family Studies, School of Human Ecology, University of Wisconsin-Madison, Madison, WI, United States; ^2^Center for Psychedelic Drug Research and Education, College of Social Work, The Ohio State University, Columbus, OH, United States; ^3^Center for Psychedelic and Consciousness Research, Department of Psychiatry and Behavioral Sciences, Johns Hopkins University, Baltimore, MD, United States

**Keywords:** 5-methoxy-N, N-dimethyltryptamine, 5-MeO-DMT, reactivation, flashback, wellbeing

## Abstract

**Background:**

The psychedelic 5-MeO-DMT has shown clinical potential due to its short duration and ability to induce mystical experiences. However, a phenomenon known as “reactivations” (similar to “flashbacks”) is a poorly understood and frequently reported phenomenon which appears associated with 5-MeO-DMT use and warranted further investigation.

**Aims:**

This study examined whether differences in age, gender, education, lifetime use, use location, and preparation strategies predict reactivations (primary outcome). Additionally, we explored how reactivations were perceived by survey respondents and whether demographic data predicted emotional valence (secondary outcome) of reported reactivations.

**Materials and methods:**

This study used secondary quantitative data from a survey assessing epidemiological and behavioral associations of 5-MeO-DMT use in non-clinical settings (*N* = 513). Descriptive statistics, chi-square tests, *t*-tests, and logistic regressions were utilized to explore aims.

**Results:**

Being female, older at the time of first 5-MeO-DMT dose, having higher educational attainment, and dosing in a structured group setting were associated with increased odds of reporting a reactivation event. Higher mystical experience scores, greater personal wellbeing and having had a non-dual awareness experience that was not substance-induced were associated with higher likelihood of reporting a neutral or positive emotional valence of a reactivation event.

**Conclusion:**

These findings suggest that reactivation phenomena, in this particular sample may most often represent a neutral or positive byproduct of the acute 5-MeO-DMT experience. More information is needed to best identify individuals most likely to experience a reactivation as a negative event to prevent such potential challenging outcomes.

## Introduction

Recent years have seen renewed interest in the use of psychedelic substances for treating various psychiatric conditions. Psilocybin has been most extensively studied, with small-scale academic studies reporting efficacy for depression and anxiety (1–9), and substance use disorders (10–14). Despite these promising results, a challenge facing the clinical uptake of psilocybin and other agents with protracted acute psychedelic effects is the amount of time and clinical resources a well conducted therapy session entails (15). This may represent a logistical and financial roadblock to making this type of therapy available in an affordable way, especially for underserved populations, highlighting the need to identify alternative options that could elicit a shorter acute psychedelic effect while maintaining comparable long-term therapeutic benefits.

One short-acting psychedelic that has the potential to fill this role is 5-methoxy-N,N-dimethyltryptamine (5-MeO-DMT). 5-MeO-DMT is a non-selective serotonin agonist with high affinity for the 5-HT1A receptor, and less affinity for the 5-HT2A and 5-HT2C receptor subtypes (16, 17). 5-MeO-DMT is found in numerous plants and in large quantities in the defense secretions of the *Incilius alvarius* toad, endemic to the Sonoran Desert (18–22). 5-MeO-DMT induces a much shorter altered-state experience, on the order of 20–60 min (23, 24), as opposed to ∼6 h for psilocybin, ∼12 h for LSD (25) and ∼8–12 h for mescaline (26). Preliminary evidence indicates that 5-MeO-DMT has the potential to ameliorate mental health disorders and improve wellbeing (21, 27). A recent survey on the epidemiology of 5-MeO-DMT showed that many respondents reported improvements in anxiety, depression, substance misuse and posttraumatic-stress-disorder (15, 27) following 5-MeO-DMT use in naturalistic settings.

Pre-clinical data offer evidence of 5-MeO-DMT’s anti-inflammatory, neuro-regenerative and anti-addictive potential through its interaction with Sigma 1 receptors, as well of its effects on glutamate receptors (16, 17, 28), both of which may have relevance in the treatment of mental health disorders. Moreover, despite the significantly reduced period of psychedelic effects compared to most other psychedelic compounds in development, a low to moderate dose of 5-MeO-DMT reliably induced a mystical experience of similar intensity to a high-dose of psilocybin administered in clinical settings (29). This is of therapeutic significance given that the occurrence of mystical experiences has been consistently associated with positive long-term outcomes (5, 14, 30–34). Taken together, these findings point to the potential role of 5-MeO-DMT as a therapeutic tool, and a viable alternative to circumvent the challenges inherent in the clinical use of longer-acting psychedelics.

However, epidemiological data (15), as well as anecdotal reports in online forums and social media groups (35, 36) indicate that the use of 5-MeO-DMT is associated with the frequent occurrence of a complex and not well understood phenomenon that has been termed “reactivation.” The term reactivation is similar to the 1960’s term “flashback,” which is defined as “a reexperiencing of certain elements of the drug induced state *after* the drug’s effects have worn off” (37, 38).

Descriptions of flashbacks include perceptual, somatic, or emotional sensations that were first experienced during the acute psychedelic state. These transient after-effects have been described as ranging from delusions to pleasant bodily sensations and perceptual illusions, to feelings of serenity, relaxation, and a sense of being “one with the world” (39).

The DSM-5 includes a similar phenomenon by the name of Hallucinogen Persisting Perceptual Disorder (HPPD) (40), and it distinguishes two subtypes of HPPD: Type 1 and Type 2. Type 1 is characterized by brief, benign, intermittent re-experiences of some aspect of the altered state induced by the psychedelic that may appear days or months after the psychedelic was taken (40). Type 2 is a radically different condition in which the re-experiencing of some aspect of the altered state generates significant distress in terms of individual, familial, social, or occupational areas of functioning on an on-going basis (41).

Flashbacks were a contributing factor to the 1970’s political banning of psychedelics (42). As such, the reported incidence of flashback-like reactivation phenomenon associated with 5-MeO-DMT use, and the phenomenon’s unknown emotional valence could potentially hinder 5-MeO-DMT’s viability as a therapeutic modality.

To help advance future clinical applications of 5-MeO-DMT, and to better understand and characterize reactivations, this study aimed to examine prevalence rates as well as predictors associated with this phenomenon. Additionally, we conducted an initial exploration of the phenomenon’s emotional valence (i.e., whether it was perceived as a positive, neutral, or negative experience).

Demographic variables (i.e., educational attainment, age at first dose, gender) and variables related to the intensity of past drug use (i.e., lifetime use, frequency of dose) used in the present study were chosen based on a review of the literature on the flashback phenomenon from the 1970’s, (37–39).

The variables grouped under the header of “preparation” in Tables 3, 4 were selected based on the idea that the longer one planned ahead for a 5-MeO-DMT experience, the more mindful and engaged in a process of self-directed positive growth one was, and wanted to see if this may be associated with reactivation experiences. The harm reduction, or as they have been called in the literature “benefit enhancement strategies” (43) of setting an intention, meditating prior to the experience, obtaining drug from a trusted source, and abstaining from using other drugs were also selected as indicators of a deliberate thoughtful process of carefully preparing for the 5-MeO-DMT experience and wanted to explore if use of these strategies was associated with an increased likelihood of reactivation experiences, and their reported emotional valence.

The variables included under the header of “salience of experience” were chosen based on the preliminary idea that higher scores on the Mystical Experiences Questionnaire, as well as higher ratings of personal meaning and life satisfaction as measured by the Persisting Effects Questionnaire items included would be predictive of higher likelihood of reactivation experiences and positive/neutral emotional valence.

The variable assessing a person’s tendency toward experiencing non-drug induced altered states (non-dual sober) was included based on the observation by Heaton and Victor (37) that flashbacks may be caused “not by psychedelic drugs but by the tendency of some drug users to mislabel and selectively attend to aspects of naturally occurring altered states of consciousness which are reminiscent of psychedelic drug states.” In this study we wanted to explore if someone that tends to experience non-dual states of awareness without using substances, would report a higher likelihood of reactivation experiences. Lastly, the variable that assesses quality of life was included to examine how the incidence of reactivation events may be associated with perceived satisfaction with life.

## Materials and methods

### Study procedure

This study used secondary data from a survey assessing epidemiological and behavioral associations of 5-MeO-DMT use in non-clinical settings (15). This study was deemed exempt from IRB review from Bowling Green State University. Each respondent was presented with a consent document, a statement regarding the purpose of the study, and eligibility criteria after clicking the link for the secure survey (hosted on surveygizmo.com). No identifying information was collected. Recruitment took place from April 2017 to August 2017. Survey respondents were English-speaking individuals who had used 5-MeO-DMT at least once during their lifetime. All survey participants in the parent study who reported using synthetic 5-MeO-DMT were eligible for this study. Potential respondents came from two different subsamples recruited at the same time:

*Subsample 1 (“Structured group”)* was recruited using an email distribution list of people in the US who used 5-MeO-DMT in a structured ceremonial group context. A detailed description of this subsample is documented elsewhere (27). Briefly, the group used laboratory-tested synthetic 5-MeO-DMT and administered it following procedures that are similar to those used in research studies with psychedelics (i.e., preparation, integration). An administrator of the group’s email distribution list sent an email with a recruitment notice for the online survey.

*Subsample 2 (“General population”)* was recruited using online advertisements and consists of individuals from the general population who reported using 5-MeO-DMT in non-structured settings (e.g., their home/apartment, outdoors). A detailed description of this sample is documented elsewhere (15). In brief, survey advertisements were posted on sites such as bluelight.org, erowid.org, and 5meodmt.org.

The present study includes 344 respondents from subsample 1 and 216 respondents from subsample 2. After excluding respondents with missing data, our final analytic sample consisted of a total of 513 respondents (Structured group subsample *n* = 344; General population subsample *n* = 169). The full survey and data are available upon request.

### Measures

Primary and secondary outcome measures for the current study were prevalence of reactivation events and emotional valence of these events when they occurred. To assess these outcomes, the following questions were administered:

*-Have you had a spontaneous re-experiencing or re-activation of a past 5-MeO-DMT experience after using the medicine (e.g., a flashback or a feeling as though your 5-MeO-DMT experience is happening again? (No/Yes)*. Those who answered yes, were further asked:
*-Was it a negative, neutral, or positive experience? (Negative vs. Neutral/Positive)*.

Predictors of reactivation and emotional valence included demographic information, preparation steps, use patterns, mystical experience phenomenology, and meaningfulness, propensity to experience non-dual states, and personal wellbeing. Respondents self-reported their demographic information including age, sex (male, female), age at first 5-MeO-DMT dose, (range: 20–50) and education (High school/Some college vs. bachelor’s or higher).

Preparation steps individuals took prior to 5-MeO-DMT administration were assessed with an item asking, *“How far in advance do you typically plan before a 5-MeO-DMT session?”* Originally recorded categorically (I do not plan, a few days, a week, a month, or more) was coded numerically as 0, 3, 7, and 30, respectively, for the regression analyses. The use of a series of benefit enhancement strategies (43) were also assessed, including *setting an intention for the 5-MeO-DMT session, meditating prior to the session, obtaining 5-MeO-DMT from a trusted source, and abstaining from alcohol and other drugs prior to the 5-MeO-DMT session* (No/Yes).

Data regarding 5-MeO-DMT use patterns were originally recorded as categorical variables: Lifetime number of 5-MeO-DMT doses (1–2, 3–4, 5–10, 11+), frequency of dose (only once, about once per year, less than once per month but more than once per year, about once per month) but were transformed to continuous variables for regression analyses by taking the floor of each category. Re-dose frequency (i.e., taking a second dose of 5-MeO-DMT within a single session) was also assessed (No/Yes). An item asking “*When was the last time you used 5-MeO-DMT*?” (Last use), originally recorded as a categorical variable (within the past month, between one and six months ago, between 6 and 12 months ago, more than 12 months ago) was coded as 0 for the category “within the last month” and using the floor option for the rest of the categories, (i.e., 1 for within one and 6 months, 6 for within 6 and 12 months) for regression analyses.

Mystical-type experiences were assessed using the Mystical Experiences Questionnaire (MEQ30), a valid and reliable 30-item self-report measure (44). The MEQ30 is scored using a 6-point Likert scale that ranges from “None, not at all” to “extreme.” For this study, we used a total MEQ mean score. Higher scores indicate stronger mystical experience. Internal consistency of the total scale was excellent (Cronbach’s alpha = 0.97).

Perceived impact of the 5-MeO-DMT experience on personal wellbeing was assessed with the question “*Do you believe that your first experience with 5-MeO-DMT and your contemplation of that experience have led to change in your current sense of personal wellbeing or life satisfaction?*” This item was scored with a 7-point Likert scale ranging from “Increased very much” to Decreased very much” (range −3 to 3). Meaningfulness of the experience was assessed with the question *“Overall, how personally meaningful was your first experience with 5-MeO-DMT?”* This item was scored with an 8-point Likert scale ranging from “No more than routine, everyday experiences” to “The single most meaningful experience of my life” (range 0–7).

Propensity to experience drug-free altered states of consciousness was assessed with the question: *“Do you have any experiences with non-dual states of consciousness (e.g., transcendental, or unitary states) that were not induced by substance use?”* (No, Yes, Unsure).

Lastly, the Subjective Wellbeing and Life Satisfaction (SWLS) scale was used to measure global cognitive judgment of their life as a whole in relationship to a self-imposed ideal using five items (45). The SWLS is a 5-item instrument that is scored on a 7-point Likert scale that ranges from “Strongly disagree” to “Strongly agree” (range −3 to 3), with higher scores denoting higher levels of wellbeing and life satisfaction. Internal consistency of this scale was good (Cronbach’s alpha = 0.86).

### Statistical analysis

Data analysis for this project proceeded in five stages. First, descriptive statistics were examined. Second, chi-square tests and *t*-tests were conducted to explore differences in age, gender, education, lifetime use, use location, preparation strategies, etc., between study subsamples (*p* < 0.05 level). Third, the primary outcome variable (reactivation: yes/no) was regressed on each individual predictor variable to assess bivariate relations using logistic regression.

The secondary outcome (emotional valence) was also regressed on each individual predictor variable. Fourth, the interaction term between the predictor and study subsample (structured group vs. general population) was included in each bivariate regression to determine whether the bivariate association was moderated by the context in which 5-MeO-DMT was consumed. Emotional valence was also regressed on each predictor variable and the interaction of that predictor variable and the study sample. Fifth, reactivation and emotional valence were regressed on all 18 predictor variables simultaneously to determine which predictor variables accounted for unique variance in the outcome, while controlling for all other predictor variables in the model. Effect sizes for-significant tests and chi-square results were calculated using the Cohen’s *d* statistic and Cramer’s *v* statistic, respectively. Given the exploratory nature of this study no adjustments were made for multiple comparisons. Analyses were conducted using Stata 15 (46).

## Results

### Descriptive statistics

The demographic characteristics of participants are shown in Table 1. On average, study participants were 43 years old, about two thirds of the sample were male. Twenty nine percent of participants were between 18 and 29 years old when they first tried 5-MeO-DMT and about 65% had a bachelor’s or higher educational degree. Effect sizes for variables that were significantly different between subsamples (structured group vs. general population subsamples) are noted on Table 1.

**TABLE 1:** Demographic characteristics of study participants.

Characteristic	Total sample *n* = 513 M (SD) or %	Structured group *n* = 344 M (SD) or %	General population *n* = 169 M (SD) or %	*P*-value *t*-test/Pearson *X*^2^	Cohen’s Cramer’s *v*
Age	43.8 (14.0)	47.8 (13.3)	35.7 (11.86)	<0.001	−0.94
Sex				<0.001	0.3
Male	66.2	54.6	89.7		
Female	33.7	45.3	10.0		
Age at first dose				<0.001	−1.3
18–29	29.2	10.7	66.8		
30–39	24.5	28.4	16.5		
40–49	21.8	26.7	11.8		
50+	24.3	34.0	4.7		
Highest education				<0.001	−0.7
High school or less	8.3	4.3	16.5		
Some college, no degree	21.2	15.1	33.7		
Associate’s degree	5.4	4.9	6.5		
Bachelor’s degree	34.5	39.2	24.5		
Advanced degree (MA/MS, PhD, MD)	30.4	36.3	18.3		

As Shown in Table 2, context of 5-MeO-DMT use (structured group subsample vs. general population subsample) was associated with numerous between-group differences, including differences in the prevalence of reactivation events. In the structured group subsample, 73% of participants reported having a reactivation event. In the general population subsample, 27% of participants reported having a reactivation event. In the structured group subsample, 86% of respondents reported their reactivation event as positive, 10% as neutral and 4% of respondents reported their reactivation event as negative. In the general population subsample 73% indicated their reactivation event had been positive, 20% said it was neutral and 7% reported their reactivation event had been negative.

**TABLE 2:** Distribution of variables by group (*n* = 513).

Independent variables	Structured group *n* = 344			General population *n* = 169		
		
	*Mean/%*	*SD*	*Range*	*Mean/%*	*SD*	*Range*
**Preparation**						
Planned time ahead (days)	22.29	11.89	0–30	5.13	8.66	0–30
Set an intention for the experience	0.88	0.32	0–1	0.60	0.49	0–1
Meditated prior to the experience	0.55	0.49	0–1	0.33	0.47	0–1
Obtained drug from a trusted source	0.91	0.27	0–1	0.66	0.47	0–1
Abstained from using other drugs	0.68	0.46	0–1	0.41	0.49	0–1
**5-MeO-DMT use patterns**						
Lifetime number of doses	3.16	3.05	1–11	5.97	3.87	1–11
Frequency of dose	1.93	0.80	1–4	2.52	0.74	1–11
Re-dose frequency*	0.51	0.50	0–1	0.52	0.50	0–1
Last use (months since last use)	6.28	5.3	0–12	7.52	5.38	0–12
**Salience of 5-MeO-DMT experience**						
Mystical Experience Questionnaire	4.29	0.90	0–5	3.41	1.25	0.1666–5
PEQ–wellbeing	2.33	1.01	−3–3	1.53	1.19	−3–3
PEQ–meaningfulness	5.79	1.05	0–7	4.56	1.91	0–7
**Other**						
**Non-dual experience while sober**						
Yes	0.66	0.47	0–1	0.40	0.49	0–1
Unsure	0.15	0.36	0–1	0.20	0.40	0–1
Satisfaction with life scale	1.11	1.35	−3–3	0.30	1.44	−3–3

*A second dose within single 5-methoxy-N,N-dimethyltryptamine (5-MeO-DMT) session. PEQ, Persistent Effects Questionnaire. *T*-test/Pearson *X*^2^ *p*-value is < 0.001 on all variables except “Re-dose frequency” (0.847), and “Last use” (0.014).

**TABLE 3:** Relationships between independent variables and occurrence of reactivation following use of synthetic 5-methoxy-N,N-dimethyltryptamine (5-MeO-DMT) in naturalistic settings (*n* = 513).

Variables	Bivariate effects			Multivariate effects		
		
	*OR*	*95% CI*	*P-value*	*OR*	*95% CI*	*P-value*
**Demographics**						
Sex (ref = male)	3.16	2.11–4.75	0.000	1.82	1.11–2.99	0.016
Age at first dose (ref = younger age)	1.05	1.03–1.06	0.000	1.00	0.97–1.02	0.967
Education (ref = lower education)	2.47	1.70–3.59	0.000	1.41	0.88–2.83	0.150
**Context of use**						
Study population (ref = general population)	7.43	4.90–11.24	0.000	2.28	1.19–4.39	0.013
**Preparation**						
Planned time ahead (days)	1.06	1.04–1.07	0.000	1.02	1.00–1.04	0.006
Set an intention for the experience (ref = no)	2.58	1.67–4.00	0.000	0.77	0.42–1.43	0.419
Meditated prior to the experience (ref = no)	2.21	1.54–3.17	0.000	1.42	0.89–2.28	0.140
Obtained drug from a trusted source (ref = no)	1.93	1.20–3.10	0.005	0.52	0.26–1.00	0.051
Abstained from using other drugs (ref = no)	2.10	1.47–3.02	0.000	1.29	0.80–2.07	0.292
**5-MeO-DMT use patterns**						
Lifetime number of doses	0.89	0.85–0.94	0.000	0.95	0.88–1.03	0.265
Frequency of dose	0.79	0.63 –0.97	0.030	1.04	0.74–1.46	0.804
Re-dose frequency*	1.16	0.81–1.64	0.404	1.17	0.74–1.87	0.488
Last use (months since last use)	0.93	0.90–0.96	0.000	0.94	0.90–0.98	0.007
**Salience of 5-MeO-DMT experience**						
Mystical Experience Questionnaire	1.98	1.62–2.42	0.000	1.26	0.97–1.63	0.082
PEQ–wellbeing	1.68	1.45–1.94	0.000	1.02	0.82–1.29	0.800
PEQ–meaningfulness	1.68	1.45–1.94	0.000	1.27	1.04–1.55	0.015
**Other**						
**Non-dual experience while sober**						
Yes	2.81	1.83–4.31	0.000	1.63	0.94–2.82	0.081
Unsure	1.94	1.12–3.37	0.017	1.67	0.86–3.24	0.129
Satisfaction with life scale	1.34	1.18–1.52	0.000	1.07	0.91–1.25	0.396

*A second dose within single 5-MeO-DMT session. PEQ, Persistent Effects Questionnaire.

**TABLE 4:** Relationships between independent variables and neutral or positive emotional valence of reactivation experiences (*n* = 295).

Variables	Bivariate effects			Multivariate effects		
		
	*OR*	*95% CI*	*P-value*	*OR*	*95% CI*	*P-value*
**Demographics**						
Sex (ref = male)	0.77	0.24–2.44	0.673	0.34	0.05–2.05	0.241
Age at first dose of 5-MeO-DMT	0.96	0.91–1.02	0.282	0.88	0.79–0.99	0.041
Education (ref = lower education)	0.92	0.24–3.50	0.908	1.67	0.19–14.07	0.636
**Context of use**						
Study population (ref = general population)	1.91	0.49–7.35	0.345	16.14	0.1.34–194.32	0.028
**Preparation**						
Planned time ahead (days)	1.00	0.95–1.04	0.954	0.97	0.89–1.04	0.450
Set an intention for the experience (ref = No)	3.22	0.92–11.22	0.066	3.10	0.45–21.34	0.250
Meditated prior to the experience (ref = No)	1.84	0.57–5.96	0.304	0.72	0.11–4.75	0.742
Obtained drug from trusted source (ref = No)	1.41	0.29–6.73	0.661	1.05	0.13–8.58	0.935
Abstained from using other drugs (ref = No)	2.10	0.66–6.72	0.207	2.03	0.32–12.72	0.449
**5-MeO-DMT use patterns**						
Lifetime number of doses	1.20	0.91–1.58	0.180	1.17	0.77–1.77	0.441
Frequency of dose	2.26	0.99–5.19	0.053	2.20	0.51–9.36	0.284
Re-dose frequency*	1.62	0.50–5.24	0.417	0.33	0.04–2.53	0.292
Last use (months since last use)	0.87	0.77–0.99	0.041	0.71	0.56–0.94	0.008
**Salience of 5-MeO-DMT experience**						
Mystical Experience Questionnaire	1.97	1.30–2.97	0.001	1.59	0.85–2.98	0.140
PEQ–wellbeing	2.06	1.40–3.02	0.000	2.86	1.49–5.48	0.002
PEQ–meaningfulness	1.12	0.71–1.75	0.617	0.67	0.34–1.30	0.246
**Other**						
**Non-dual experience while sober**						
Yes	4.90	1.43–16.77	0.011	9.32	1.20–72.10	0.032
Unsure	6.39	0.74–55.13	0.092	12.54	0.90–173.52	0.059
Satisfaction with life scale	1.01	0.65–1.58	0.930	0.94	0.55–1.60	0.833

*A second dose within single 5-MeO-DMT session. PEQ, Persistent Effects Questionnaire.

### Reactivation

Results of bivariate and multivariable logistic regression analyses for reactivation are shown on Table 3. In the bivariate analyses, being a female, older age at the time of first 5-MeO-DMT dose, higher educational attainment, and being in the structured group subsample were significantly associated with increased odds of reporting a reactivation event. Interaction tests indicated that age at first dose and time since last use were moderated by study subsample such that being older at first dose and longer time since last use was more strongly associated with higher likelihood of reporting a reactivation in the structured group subsample compared to reporting a reactivation in the general population subsample.

In the multivariable model, females were about twice as likely to report reactivation events (OR = 1.82, CI: 1.11–2.99, *p* = 0.016). Using 5-MeO-DMT in the structured group setting (OR = 2.28, CI: 1.19–4.39, *p* = 0.013), planned ahead time (OR = 1.02, CI: 1.00–1.04, *p* = 0.006) and greater meaningfulness of the 5-MeO-DMT experience (OR = 1.27, CI: 1.04–1.55, *p* = 0.015) were significantly associated with an increased likelihood of reporting a reactivation event, while longer time since the last 5-MeO-DMT use (OR = 0.94, CI: 0.90–0.98, *p* = 0.007) was associated with a decreased likelihood of reporting a reactivation event.

### Emotional valence

Table 4 shows bivariate and multivariable results of logistic regression analyses of the emotional valence attributed to the reactivation event. In the bivariate analyses longer time since the last 5-MeO-DMT use was associated with a decreased likelihood of reporting a neutral or positive reactivation event. Higher mystical experience scores, greater personal wellbeing, and having had a non-dual awareness experience that was not substance-induced were significantly associated with higher likelihood of reporting neutral or positive emotional valence of a reactivation event. The interaction tests indicated that current personal wellbeing was moderated by study subsample, such that greater wellbeing was more strongly associated with higher likelihood of reactivations being reported as neutral/positive in the structured group. In the general population there was no association between greater wellbeing and positive/neutral emotional valence.

In the multivariable model older age at first dose (OR = 0.88, CI: 79–0.99, *p* = 0.041) and longer time since last 5-MeO-DMT use (OR = 0.71, CI: 0.56–0.94, *p* = 0.008) decreased the likelihood, while being in the structured subsample (OR = 16.14, CI: 1.34–194.32, *p* = 0.028), a higher score on personal wellbeing (OR = 2.86, CI: 1.49–5.48, *p* = 0.002) and having had a non-dual experience that was not drug-induced (OR = 9.32, CI: 1.20–72.10, *p* = 0.032) significantly increased the likelihood of positive or neutral emotional valence of reactivation events. Study group was not associated with emotional valence of reactivation events.

## Discussion

To begin to examine the 5-MeO-DMT reactivation phenomenon and how it may compare to the better-known LSD-related flashback and HPPD, in the present study we conducted an initial exploratory analysis of the prevalence, predictors and emotional valence of reactivation phenomena associated with the use of synthetic 5-MeO-DMT in two separate study subsamples. We found a statistically significant difference in the rate of reactivations reported between the study subsamples (73% in the structured group subsample vs. 27% in the general population subsample). Nearly all survey respondents indicated that their reactivation experiences were positive or neutral (96% in the structured group subsample and 93% in the general population subsample). Having taken the substance in the structured group setting, and being a female showed the strongest independent effects on predicting reports of reactivation. To our knowledge, this study is the first to show that context of use and female sex may increase the likelihood of reactivation phenomena associated with synthetic 5-MeO-DMT use.

In several review papers of the LSD-related flashback phenomena this sex difference is not noted (40, 47). Furthermore, Baggott et al. (48) conducted an online survey study to document flashback phenomena in a large sample of users who reported having used LSD, psilocybin, and several other drugs, and although this sex difference was not noted there either, the authors report that almost 90% of the sample were male (compared to 68% in the present study). Therefore, it could be that when survey samples include more females this phenomenon is more apparent. Future studies should focus on exploring this topic specifically among females to better understand this phenomenon of the 5-MeO-DMT experience. The finding that neither number of lifetime doses, nor frequency of dosing increased the odds of reactivation phenomena is consistent with data from the 1970’s on the prevalence of flashbacks (37, 49).

It is notable that nearly all participants who reported a reactivation in the current study perceived it as a positive experience. This observation suggests that the reactivation phenomenon might be conceptualized not as an adverse effect, but rather as neutral or positive byproduct of the acute 5-MeO-DMT experience when administered in certain settings. It is possible that reactivations may even contribute to the antidepressant and anti-anxiety effects that have been published previously (15, 17, 21). Indeed, that the greater length of time an individual planned for their 5-MeO-DMT experience, the greater their report of meaning attributed to their 5-MeO-DMT experience, and the higher the odds of reactivation, could be indicative of an intentional self-directed behavior process facilitated by the use of 5-MeO-DMT.

In this sense, these initial findings would seem to suggest that 5-MeO-DMT reactivations may be considered to be more akin to HPPD Type 1, as opposed to Type 2 (40), but these findings are preliminary and limited by the nature of the study (i.e., selection bias potentially leading to the high rate of positive valence, and the retrospective nature of the cross-sectional survey design). Furthermore, there are anecdotal reports from individuals who do struggle with reactivations for weeks after a high-dose of toad-derived 5-MeO-DMT that have been reported in online forums (i.e., 5-Hive) which would be indicative of HPPD Type 2. Based on the few online anecdotal reports, it seems that people who are more likely to struggle with reactivations longer term are those that receive an unweighted and hence, likely higher than needed dose of 5-MeO-DMT derived from toad secretions. Unfortunately, in this study, we did not have data regarding dosage to examine whether higher doses would show higher odds of reactivations compared to lower or moderate doses.

The differential prevalence rates of reactivation among our study samples seem consistent with findings from early reports of flashback prevalence rates among psychedelic users. These early findings reported a wide range of prevalence rates, from 1 in 4 (50) to 1 in 20 (51), and from 15 to 77% of psychedelic users (52). Strassman highlights a list of methodological concerns regarding criteria for determining these prevalence rates (52).

In the multivariable model for emotional valence, greater personal wellbeing was significantly positively associated with positive or neutral emotional valence of these reactivation events. It could be that, for those that subsequently have a positive reactivation experience, they also had a positive emotional experience during the acute effects of the 5-MeO-DMT (state dependent processing), which in turn contributes to their enhanced sense of wellbeing. However, the opposite could also be true (negative experiences during the acute effects being related to interpretation of negative reactivation should it occur).

The finding that 80% of survey respondents in the structured group also reported improvements in symptoms of depression and anxiety (27), together with the present results provide a theoretical rationale for an overall benign or positive conceptualization of reactivation phenomena among people who consume synthetic 5-MeO-DMT in structured ceremonial settings. This finding further emphasizes the importance of using 5-MeO-DMT in a carefully controlled clinical setting where personal wellbeing and meaning-making are supported and facilitated, and thus positive experiences and outcomes are more likely. Nevertheless, it should be noted that, for the small percentage of those who report their reactivation is a negative experience, this may contribute to anxiety, panic, and other concerning emotional states requiring emergency crisis treatment. More information is needed to understand how to best identify those most likely to experience a reactivation as negative to prevent such challenging outcomes.

Having had a “non-dual” experience (e.g., transcendental, or unitary states) that was not induced by substance use significantly predicted neutral or positive emotional valence of reactivation events. The term “non-dual” derives from Tibetan Buddhism. It refers to a state of awareness that transcends the subject-object dichotomy which underlies internally and externally driven mentation (53). Although such dichotomies are known to be somewhat natural, psychological inflexibility about them can result in an excessively fragmented experience that leads to ruminative thought patterns which are associated with various mental health disorders (53–55).

In line with this, Davis et al. (56) report that increases in psychological flexibility mediate the relation between mystical and insightful experiences on decreases in depression and anxiety following a psychedelic experience. In terms of the neural correlates of this increase in psychological flexibility, neuroimaging studies have revealed that there is a decoupling of the various structures that make up the “default mode network” (57), a large-scale brain network believed to underlie internally focused mentation. The acute temporary decoupling of the default mode network’s structural nodes during the acute psychedelic state, along with global increases in connectivity are hypothesized to be correlated with the experience of “ego dissolution” (1, 58–60), and to result in a less constrained (higher entropy) style of cognition (1, 59). This in turn is believed to map out to psychological flexibility and mediate positive therapeutic outcomes (56, 61).

In terms of electrical activity in the brain, another neural correlate of the experience of non-dual ego dissolution is a significant decrease in *alpha* brain oscillations in the posterior cingulate cortex (62). In line with this, Acosta-Urquidi (63) also found consistent and significant *alpha* oscillations suppression in the cortex of individuals under the acute effects of 5-MeO-DMT in naturalistic settings, which was followed by an *alpha* rebound effect (a return of spectral power in this band as the effects of the drug subsided). This is interesting given that some of the most common precipitating factors of reactivation events are various activities such as falling asleep, meditating, mindfulness practices, relaxation techniques, and being in nature, all of which are known to induce *alpha* oscillations (64, 65). It is possible that reactivations could be experienced when the brain shifts from higher power brain oscillations, like *beta*, to lower power *alpha* oscillations during these types of activities. Moreover, because there is a sharp suppression of these oscillations under the effect of vaporized 5-MeO-DMT, followed by an *alpha* recovery rebound effect, it is possible that the combination of these experiences triggers a memory of the transition from the deep 5-MeO-DMT state back to normal consciousness, which is perceived or felt as a reactivation.

Of note, sex differences in modulation of *alpha* peak oscillations indicate that *alpha* oscillatory activity across time periods changes more in females than in males (66), and overall brain oscillations have been found to be highly influenced by sex differences (67), which could potentially underlie the significantly greater likelihood of reactivations reported by females in the present study. Future high-density EEG studies with 5-MeO-DMT could help elucidate the etiology of reactivations. For example, exploring whether baseline *alpha* power is associated with reactivations could be an intriguing next step.

Although as we have shown here, reactivations need not be construed as negative/adverse effects/events, it is important to note that the prevalence rates described herein are in relation to synthetic 5-MeO-DMT administered *via* vaporization route (i.e., smoked). It is possible that the rapid onset of the 5-MeO-DMT state afforded by this route of administration contributes to the likelihood of reactivation experiences. Indeed, a recent study (68) found lower rates of reactivation among a subsample of individuals who use 5-MeO-DMT *via* intramuscular (IM) injection. 5-MeO-DMT administered *via* IM injection likely produces a more gradual experience that may be physiologically and psychologically easier to integrate and thus less likely to promote reactivation phenomena. However, such findings have not been explored using laboratory assessments and thus further research is needed to determine the likelihood of reactivation following various routes of administration of 5-MeO-DMT in laboratory settings.

Interestingly, if 5-MeO-DMT were to be moved along the FDA pipeline to be tested as a pharmacological treatment for depression, or substance use disorders, it would most likely be administered *via* IM injection or intranasal formulation, not unlike ketamine treatment for depression, (69), which also has a slower onset compared to vaporization, thus potentially decreasing the likelihood that reactivations are experienced. Given that the data presented here indicate that females have a significantly higher likelihood of reporting reactivations, perhaps certain precautions can be taken to reduce the potential for any adverse effects resulting from unanticipated reactivation experiences. This in turn, however, could prime participants to be more likely to experience reactivations given the role that expectancy bias has been noted to have in association with the flashback phenomena (70). In fact, the rate of reactivations being so much higher among those in the structured group subsample could be hypothesized to be a function of expectancy bias, given that those who partake of 5-MeO-DMT in the structured group undergo an orientation session where the possibility of experiencing reactivations post 5-MeO-DMT is addressed, whereas those in the general population sample may have not had such expectancies.

Importantly, a gap in the literature exists with regard to characteristics of the reactivation phenomena that warrants further investigation: how long does each transient episode last? In the cases when the emotional valence of reactivations is negative, how disruptive are they and how often do they occur? How long after 5-MeO-DMT use do reactivations tend to occur? What are some of the precipitating triggers individuals may report?

Interestingly, anecdotal reports online do seem to suggest that reactivations tend to occur mainly at night as people are drifting off to sleep, and generally tend to dissipate the longer the time has elapsed from the time they took 5-MeO-DMT. In a broad sense, it appears that reactivations are experienced as transient but intense (Type 1 HPPD) and tend to subside days or weeks after 5-MeO-DMT use in most cases, as opposed to persisting over longer periods of time as is the case with HPPD Type 2, but much more data is needed in order to ascertain this confidently. Nevertheless, as noted above, there are some anecdotal reports in the popular literature (i.e., Facebook, 5-Hive forum) that document instances of people experiencing anxiety and impaired sleep from continued reactivation experiences months after the experience. Mixed methods research designs that can collect qualitative data regarding reactivation details in upcoming clinical trials will be very valuable to enhance our understanding of the reactivation phenomena and how it may compare to HPPD Type 1 and 2.

Several limitations of this study warrant consideration. First, the cross-sectional nature of this study precludes the ability to infer causality. There was no 5-MeO-DMT dosage information available, and the route of intake (inhalation) likely delivered non-standardized doses across participants. Survey studies are further limited by the retrospective nature of reporting, which is subject to recall bias (71). As noted above, this study is further limited by selection bias. Respondents who have had negative reactivations may be less likely to have wanted to complete the survey, while those who participated in this survey may be inclined to report positive associations due to having a favorable view of the substance, which could explain the high rates of positive valence reported herein. While in the structured group, 5-MeO-DMT was tested for purity, there is no assurance that was done among the general population users, which may account for different rates of reactivation.

Finally, although we used the only empirical epidemiological dataset on patterns of 5-MeO-DMT use available to date and showed that negative experiences of reactivation are highly unlikely, anecdotal reports in popular media (e.g., Facebook, Reddit) have documented examples of individuals who experience persistent, unpleasant reactivations. It is important to clarify that these reports result from the use of *I. alvarius* toad-derived 5-MeO-DMT, rather than synthetically produced 5-MeO-DMT. Importantly, a mixed methods study incorporating qualitative reports about reactivation’s precipitating factors and the phenomenology of the felt sense of experience and its emotional valance could help determine if reactivations are more likely to be challenging when consuming toad-derived, compared to synthetically produced, 5-MeO-DMT.

In conclusion, our findings show that the occurrence of reactivation phenomena resulting from synthetic 5-MeO-DMT use is not uncommon. Reactivation experiences are largely perceived as positive or neutral. Rather than being construed as adverse effects, they may well be a contributing factor to long-term therapeutic benefits. Future prospective clinical studies exploring synthetic 5-MeO-DMT as a potential treatment for psychiatric conditions need not be deterred based on the known occurrence of this not-yet well understood phenomena. As clinical research programs with 5-MeO-DMT commence, it will be crucial to collect data that can help further characterize reactivations so they can be harnessed to support personal growth processes of psychological change and wellbeing, and prevent, to the extent possible any potential negative effects from reactivation events.

## Data availability statement

The data is available by request. Inquiries can be directed to AKD, davis.5996@osu.edu.

## Ethics statement

The studies involving human participants were reviewed and approved by Bowling Green State University. The patients/participants provided their written informed consent to participate in this study.

## Author contributions

AMOB conceptualized the study and research design, conducted the secondary data analyses, and wrote the manuscript. CLR made a substantial contribution to the conceptualization, research design, interpretation of data, and edited the manuscript. RLL made a substantial contribution to the early conceptualization of the research project as well as to the conceptualization, data collection, and authoring of the primary data set out of which this secondary data analysis derives. AKD made substantial contributions to the conceptualization, methodology, formal analysis, investigation, resources, data curation, writing—original draft, writing—review and editing, project administration, funding acquisition, and supervision. All authors contributed to the article and approved the submitted version.
